# TRAF4 promotes lung cancer aggressiveness by modulating tumor microenvironment in normal fibroblasts

**DOI:** 10.1038/s41598-017-09447-z

**Published:** 2017-08-21

**Authors:** EunGi Kim, Wanyeon Kim, Sungmin Lee, Jahyun Chun, JiHoon Kang, Gaeul Park, IkJoon Han, Hee Jung Yang, HyeSook Youn, BuHyun Youn

**Affiliations:** 1 Department of Integrated Biological Science, Pusan National University, Busan, 46241 Republic of Korea; 2 Department of Biological Sciences, Pusan National University, Busan, 46241 Republic of Korea; 3 Department of Integrative Bioscience and Biotechnology, Sejong University, Seoul, 05006 Republic of Korea; 4 Department of Biology Education, Present Address: Korea National University of Education, Cheongju, 28173 Republic of Korea

## Abstract

Normal fibroblasts surrounding tumor cells play a crucial role in cancer progression through formation of the tumor microenvironment. Because factors secreted from normal fibroblasts can modulate the tumor microenvironment, it is necessary to identify key factors associated with regulation of secreted factors and to investigate the molecular mechanisms contributing to the tumor microenvironment formation process. In this study, we found that radiation induced the expression and K63-linkage poly-ubiquitination of TRAF4 in normal lung fibroblasts. The K63-linkage poly-ubiquitinated TRAF4 formed complexes with NOX2 or NOX4 by mediating phosphorylated p47-phox in normal lung fibroblasts. Moreover, we showed that TRAF4 stabilized NOX complexes by decreasing lysosomal degradation of NOX2 and NOX4 after irradiation. NOX complexes increased endosomal ROS levels that were permeable into cytoplasm, leading to NF-κB-mediated ICAM1 up-regulation. Soluble ICAM1 was subsequently secreted into conditioned media of radiation-activated normal lung fibroblasts. The conditioned media from irradiated normal fibroblasts enhanced proliferation and epithelial-mesenchymal transition of non-small cell lung cancer cells both *in vitro* and *in vivo*. These results demonstrate that TRAF4 in irradiated fibroblasts is positively associated with aggressiveness of adjacent cancer cells by altering the tumor microenvironment. Thus, we suggest that regulation of TRAF4 might be a promising strategy for cancer therapy.

## Introduction

Tumor stroma promotes cancer progression by regulating the microenvironment surrounding cancer cells. The tumor stroma consists of basement membrane, immune cells, capillaries, fibroblasts, and extracellular matrix (ECM)^1^. It has been suggested that interaction between cancer cells and the tumor microenvironment is the driving force that enhances proliferation, migration, and invasion of cancer cells. In particular, fibroblasts are reprogrammed by several factors secreted from nearby cancer cells known as cancer-associated fibroblasts (CAFs). CAFs, which are the most abundant cells in the tumor microenvironment, enhance cancer malignancy and metastasis^2^. CAFs have been reported to be involved in cancer progression through the secretion of growth or pro-inflammatory factors including TGF-β, HGF, and CXCL12^3^. In addition, EDA-fibronectin, α-SMA, and Tenascin C are commonly up-regulated in CAFs and have the potential for use as functional and diagnostic markers^3^. Given that elucidation of the molecular mechanisms of the cancer-CAF interaction may provide new insight into the development of cancer therapy, it is important to investigate the signaling pathway responsible for normal fibroblasts to CAFs conversion.

The tumor necrosis factor receptor-associated factor (TRAF) family intensifies immune responses by mediating the signaling pathways from tumor necrosis factor receptors and interleukin-1/Toll-like receptors to downstream effectors^4^. TRAF4 is a member of the TRAF family that is known to play roles in developmental steps related to neural tube closure, axial skeleton formation, and tracheal ring formation^5^. Notably, TRAF4 is commonly overexpressed in various types of cancers including breast, lung, ovary, colon, and prostate cancer^6^. Overexpression of TRAF4 is associated with enhancement of tumor proliferation, invasion, and migration^7, 8^. Recently, TRAF4 was reported to activate TGF-β signaling and transduce both SMAD and non-SMAD pathways to promote breast cancer development^9^. Moreover, K63-linkage poly-ubiquitination of TRAF4 was shown to result in increased TRAF4 activity as an E3 ubiquitin ligase to mediate protein stabilization of TβRI through ubiquitination-dependent degradation of SMURF2, contributing to tumor malignancy^9^.

The nicotinamide adenine dinucleotide phosphate (NADPH) oxidase (NOX) family, which has catalytic subunits and transmembrane proteins, consists of five subfamilies, NOX1 to NOX5^10^. Functional NOXs reportedly occur in a complex with upstream activators or organizer subunits (p22-phox, p47-phox, p67-phox, and p40-phox) and guanosine triphosphatase (GTPase) Rac1^11^. In general, NOXs in a complex work as electron acceptors and produce reactive oxygen species (ROS) including superoxide (O_2_^−^) through electron transfer reactions^10^. Several studies have reported a role of ROS as signaling molecules in the induction of tumor proliferation, angiogenesis, and tumor malignancy^12–14^. However, the contribution of the NOX complex signaling pathway to creation of a tumor microenvironment in stroma cells remains largely elusive.

To widen the comprehension of normal fibroblasts as CAFs, we focused on the role of TRAF4 in modulating the tumor microenvironment in normal lung fibroblasts. We found that radiation induced the expression of TRAF4 and detected the involvement of TRAF4 in the production of endosomal ROS by a NOX complex in normal lung fibroblasts. Furthermore, we investigated whether TRAF4/NOX complex-mediated endosomal ROS promotes secretion of soluble ICAM1 (sICAM1) to enhance tumor progression. Based on our results, we suggest that the TRAF4/NOX complex is a key regulator involved in alteration of the tumor microenvironment in normal lung fibroblasts.

## Results

### TRAF4 is up-regulated and K63-linkage poly-ubiquitinated in normal lung fibroblasts in response to irradiation

**Figure 1:**
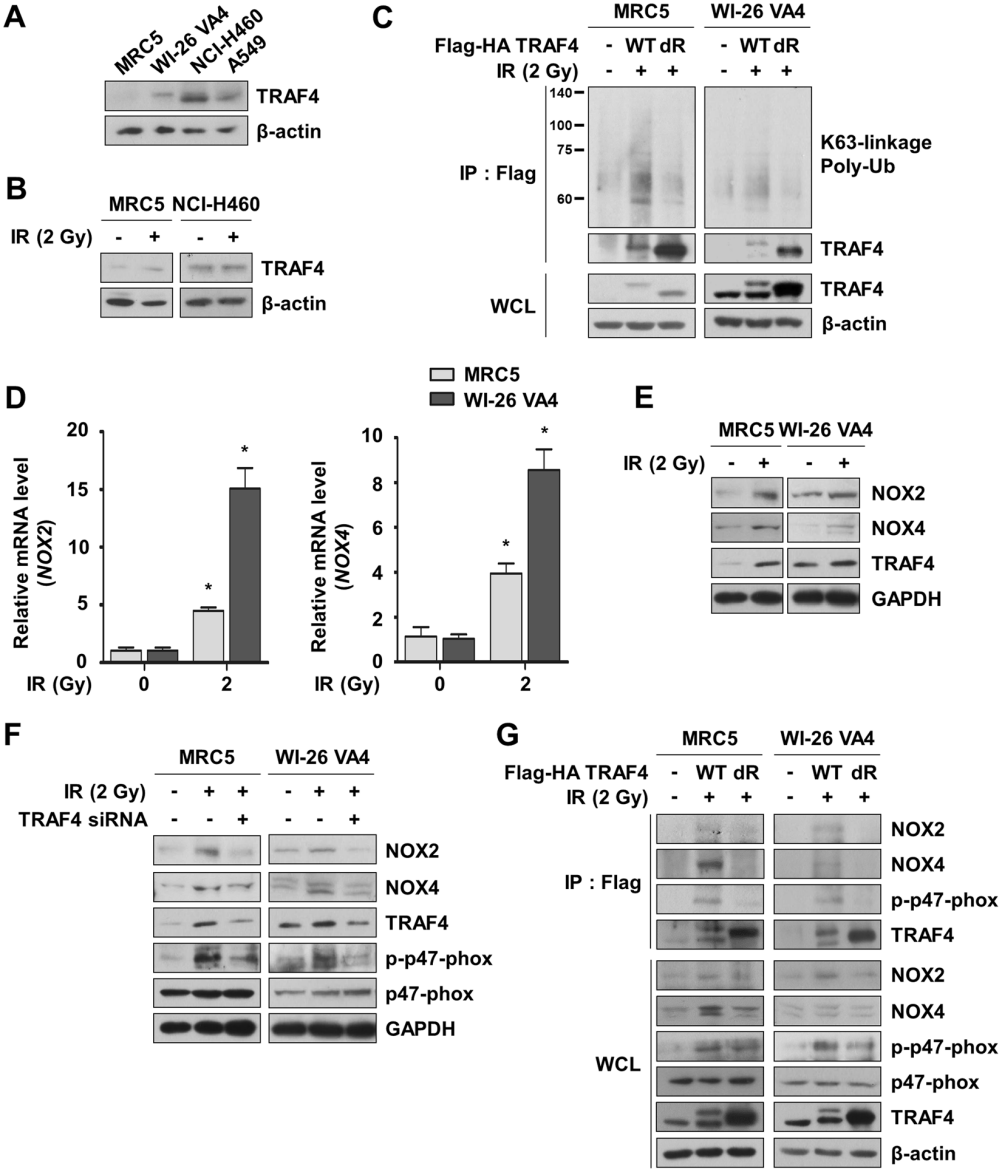
Radiation induced expression and activation of TRAF4 and complex formation of TRAF4 with NOX2 and NOX4 in normal lung fibroblasts. (**A**) Expression of TRAF4 in normal lung fibroblasts and NSCLC cells was assessed by Western blotting. (**B**) Radiation-induced TRAF4 protein expression was detected by Western blotting. Cell lysates were prepared after irradiation (2 Gy, 4 h). (**C**) Radiation-induced K63-linkage poly-ubiquitination of TRAF4 was measured by an ubiquitination assay. Cells were transfected with mock, Flag-HA-TRAF4 WT, or Flag-HA-TRAF4 dR mutant. Cell lysates were immunoprecipitated with anti-Flag antibody and subsequently analyzed by Western blotting with anti-K63-linkage poly-ubiquitin (Ub) antibody (WCL, whole cell lysates). (**D**) Radiation-induced mRNA expression of NOX2 and NOX4 was assessed by qRT-PCR. Cells were harvested after irradiation (2 Gy, 3 h). Error bars, ± SEM (n = 3); ^*^ *p* < 0.05 compared with non-irradiated cells. (**E**) Radiation-induced protein expression of NOX2 and NOX4 was measured by Western blotting. Cell lysates were prepared after irradiation (2 Gy, 4 h). (**F**) Effects of TRAF4 knockdown on expression of two NOXs and phosphorylation of p47-phox was assessed by Western blotting. (**G**) To determine NOX complex formation in response to radiation, interactions between TRAF4 and NOX2, NOX4, or phosphorylated p47-phox were investigated by IP assay.

TRAF4 is a key factor involved in regulation of inflammation and the innate immune system in normal tissue^15, 16^, as well as in the progression of breast cancer through its interplay with TβRI signaling^9^. However, the role of TRAF4 in response to irradiation remains largely elusive. To analyze the basal levels of TRAF4 in normal lung cells and NSCLC cells, we measured the expression of TRAF4 in normal lung fibroblasts (WI-26 VA4 and MRC5 cells) and NSCLC cells (A549 and NCI-H460 cells). We found that protein levels of TRAF4 are up-regulated in NSCLC cells relative to normal lung fibroblasts (Fig. 1A). Irradiation increased protein levels of TRAF4 in MRC5 cells, but did not affect TRAF4 expression in NCI-H460 cells (Fig. 1B). Based on this information and our results, we aimed to demonstrate the function of radiation-induced TRAF4 expression in normal lung fibroblasts. In a previous study, TGF-β activated the E3-ligase of TRAF4 through K63-linkage poly-ubiquitination, resulting in TRAF4-mediated stabilization of TβRI and promotion of TβRI signaling in breast cancer cells^9^. We next determined whether irradiation induces K63-linkage poly-ubiquitination of TRAF4 to investigate activation of TRAF4 in response to irradiation. We observed radiation-induced K63-linkage poly-ubiquitination in TRAF4 wild-type (WT), but not in mutant TRAF4 (dR; deletion of RING domain) (Fig. 1C). In addition, TRAF4 was found to interact with TβRI and SMURF2 and to activate TβRI signaling by phosphorylation of SMAD3 and p38 (Supplementary Fig. 1). These results suggest that TRAF4 is up-regulated and activated through K63-linkage poly-ubiquitination by irradiation in normal lung fibroblasts.

### NOX2 and NOX4 are up-regulated by irradiation and subsequently interact with TRAF4

Microarray data for gene expression profiling of CAFs revealed up-regulation of NOX4, a key regulator of ROS production^17^. Because NOX2 is the prototype of the NOX family^10^, we determined whether both NOX2 and NOX4 are associated with the radiation response in normal lung fibroblasts. We showed that mRNA and protein levels of NOX2 and NOX4 increased dramatically in response to radiation in two normal lung fibroblasts (Fig. 1D,E). TRAF4 is known to induce phosphorylation of p47-phox and to interact with NOX complexes through binding of phosphorylated p47-phox^18^. Thus, we analyzed the effects of radiation and TRAF4 knockdown on the expression of NOX2 and NOX4, as well as the phosphorylation of p47-phox. As shown in Fig. 1F, protein levels of NOX2 and NOX4 and the phosphorylation of p47-phox were elevated by radiation, but were decreased by further TRAF4 knockdown. To investigate the associations of NOX complexes with K63-linkage poly-ubiquitinated TRAF4, we conducted an immunoprecipitation (IP) assay using TRAF4 WT and dR constructs. We observed interactions of NOX2, NOX4, and phosphorylated p47-phox with TRAF4 WT in response to radiation (Fig. 1G). Taken together, these findings suggest that K63-linkage poly-ubiquitinated TRAF4 interacts with NOX2 or NOX4 mediated by phosphorylated p47-phox after irradiation in normal lung fibroblasts.

### NOX2 and NOX4 are localized to endosomes and produce endosomal ROS

**Figure 2:**
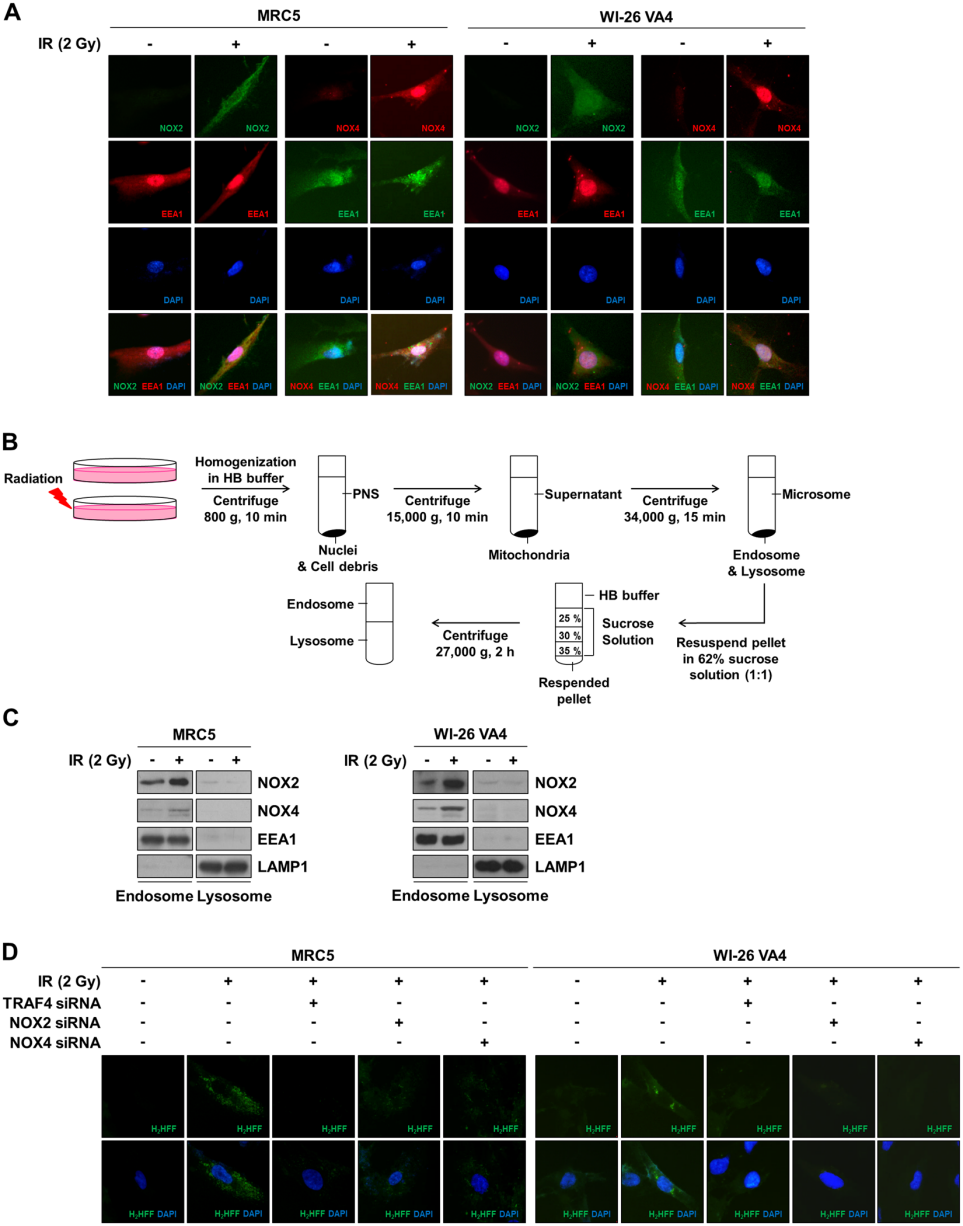
NOXs were localized in endosomes and produced ROS. (**A**) Radiation-induced subcellular localization of NOX2 and NOX4 were visualized by immunocytochemistry. EEA was used as an endosome biomarker. (**B**) An experimental scheme for endosome/lysosome fractionation was described. After irradiation, flotation-gradient fractionation was performed to isolate endosomes and lysosomes. (**C**) Protein levels of NOX2 and NOX4 in endosomes and lysosomes were investigated by Western blotting. After irradiation (2 Gy, 4 h), fractions for endosomes or lysosomes were prepared through flotation-gradient fractionation of cell lysates. Protein levels of NOX2 and NOX4 were measured by Western blotting in endosomal and lysosomal extracts. (**D**) The effects of knockdown of TRAF4, NOX2, or NOX4 on endosomal ROS were visualized with treatment of fluorescent dye H_2_HFF-BSA (Green).

NOX complexes can be located in several organelles, including the plasma membrane, endosome, ER, and nucleus, and endosomal NOX complexes are responsible for increases in ROS levels in cytoplasm^18–21^. Therefore, we focused on the localization of NOX2 and NOX4. A double immunocytochemistry assay for co-localization of NOX2 or NOX4 with endosome marker EEA1 was conducted. As shown in Fig. 2A, radiation-induced NOX2 and NOX4 were up-regulated and localized to endosomes. We also conducted flotation-gradient fractionation to isolate endosomes and lysosomes (Fig. 2B). Radiation-induced NOX2 and NOX4 were observed in endosomes, but not in lysosomes (Fig. 2C). To determine whether NOX complexes are associated with ROS generation in endosomes, we used OxyBURST green fluorescence (H_2_HFF-BSA), which specifically allows the detection of endosomal ROS production. We found that endosomal ROS levels increased in response to radiation, but decreased in response to further knockdown of TRAF4, NOX2, or NOX4 (Fig. 2D). Thus, these results indicate that NOX2 and NOX4 associated with TRAF4 lead to an increase in endosomal ROS levels in response to radiation.

### Lysosome-dependent degradation of NOX2 and NOX4 is regulated by TRAF4

**Figure 3:**
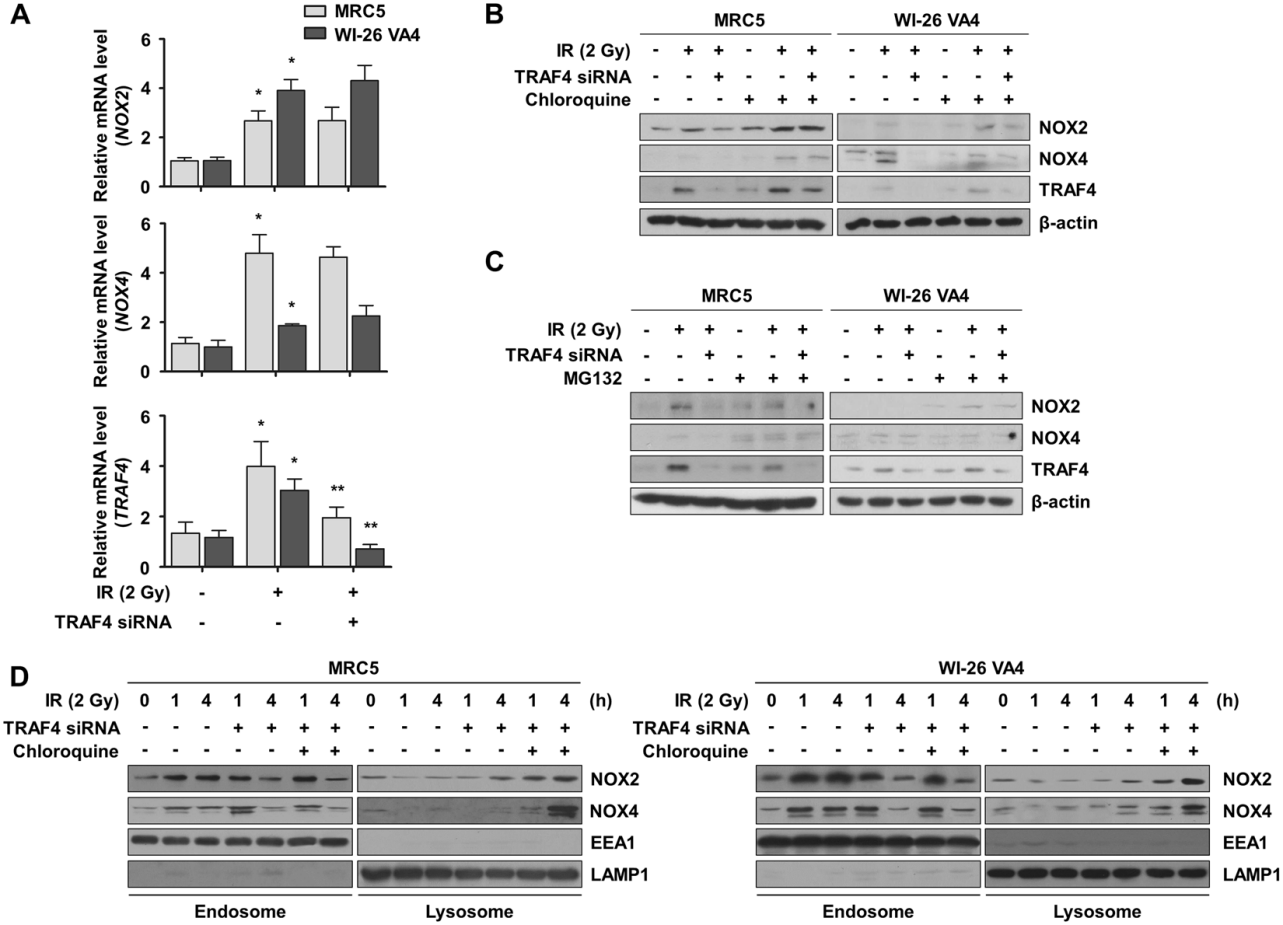
Radiation-induced NOX2 and NOX4 were stabilized by TRAF4 in endosomes. (**A**) The effects of knockdown of TRAF4 on radiation-induced mRNA levels of NOX2, NOX4, and TRAF4 were assessed by qRT-PCR. Error bars, ± SEM (n = 3); ^*^ *p* < 0.05 compared with non-irradiated cells, ^**^ *p* < 0.05 compared with irradiated cells. (**B**) Alterations of protein levels of NOX2 and NOX4 upon inhibition of lysosomal degradation were assessed by Western blotting. Cells were treated with Chloroquine, an inhibitor of lysosomal degradation, for 30 min prior to irradiation. (**C**) Alterations of protein levels of NOX2 and NOX4 upon inhibition of proteasomal degradation were assessed by Western blotting. Cells were treated with MG132, an inhibitor of proteasomal degradation, for 30 min prior to irradiation. (**D**) Effects of Chloloquine on protein stability of NOX2 and NOX4 in endosomes or lysosomes were measured by Western blotting. Endosome/lysosome fractionation was conducted after irradiation (1 h or 4 h).

Our results showed that protein levels of NOX2 and NOX4 were reduced by transient transfection of TRAF4-specific siRNA (Fig. 1F,G). To explore whether TRAF4-mediated signaling regulates the expression of NOX2 and NOX4, we measured mRNA levels of NOX2 and NOX4 in normal lung fibroblasts treated with TRAF4-knockdown. As shown in Fig. 3A, the mRNA levels of NOX2 and NOX4 were not reduced in normal lung fibroblasts treated with TRAF4-knockdown compared to control cells in response to radiation. Thus, we hypothesized that TRAF4 is associated with the regulation of NOX2 and NOX4 protein stability. It has been reported that degradation of NOX2 and NOX4 could be mediated by both proteasome and lysosome^22^. To determine whether TRAF4 regulates protein stability of NOX2 and NOX4 through proteasome- or lysosome-dependent degradation, normal lung cells were treated with a lysosome inhibitor (chloroquine) or a proteasome inhibitor (MG132) before irradiation. We showed that down-regulation of NOX2 and NOX4 mediated by TRAF4 knockdown was rescued by inhibition of lysosome, but not by inhibition of MG132 (Fig. 3B,C). These results indicate that radiation-induced NOX2 and NOX4 are stabilized by TRAF4-associated inhibition of lysosome-dependent degradation. To further investigate whether localization of NOX2 and NOX4 to the lysosome was delayed by interacting with TRAF4, we conducted endosome/lysosome isolation and measured protein levels of NOX2 and NOX4 in a time-dependent manner. Radiation-induced NOX2 and NOX4 were accumulated in endosomes for 4 h after irradiation, but diminished in endosomes and slightly observed in lysosomes by TRAF4 knockdown (Fig. 3D). In particular, we observed dramatic accumulations of NOX2 and NOX4 in lysosomes in response to TRAF4 knockdown following treatment with chloroquine. In addition, the effects of TRAF4 knockdown and chloroquine treatment on the accumulation of NOX2 and NOX4 in endosomes or lysosomes were confirmed by an immunocytochemistry assay (Supplementary Fig. 2). Collectively, these data suggest that radiation-activated TRAF4 delays lysosome-dependent degradation of NOX2 and NOX4 by maintaining localization of NOX complexes in endosomes.

### Increased endosomal ROS levels by NOX complexes are associated with NF-κB activation and subsequent ICAM1 up-regulation

**Figure 4:**
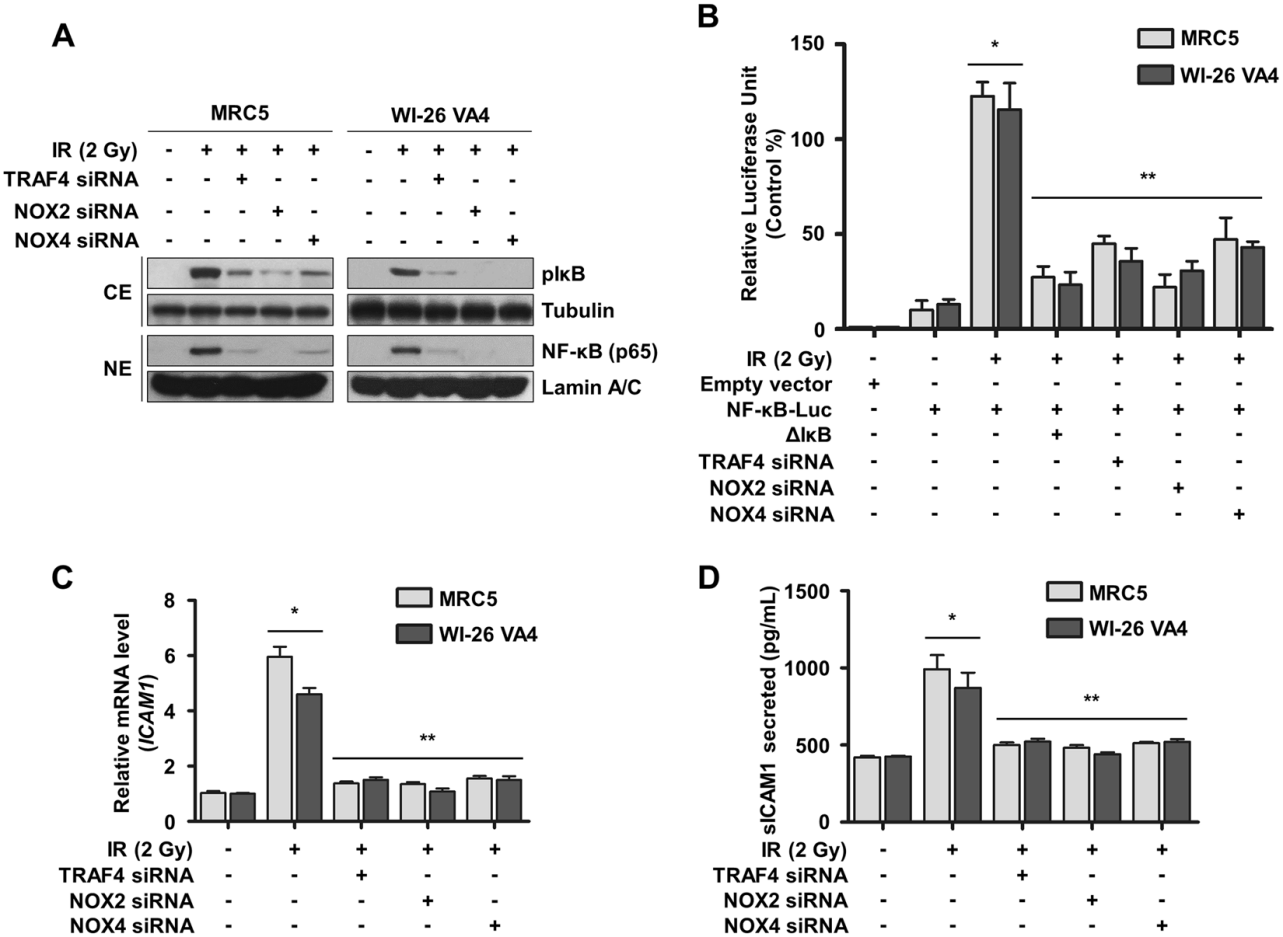
TRAF4-NOX complex induced ICAM1 expression through the NF-κB pathway in response to radiation. (**A**) The effects of knockdown of TRAF4, NOX2, or NOX4 on activation of NF-κB signaling were detected by Western blotting. Cell lysates were fractionated into cytosolic extract (CE) and nuclear extract (NE). Tubulin and Lamin A/C were used as markers for CE and NE, respectively. (**B**) Effects of knockdown of TRAF4, NOX2, or NOX4 on transcriptional activity of NF-κB were measured by a luciferase assay. Error bars, ± SD (n = 3); ^*^ *p* < 0.05 compared with non-transfected and non-irradiated cells, ^**^ *p* < 0.05 compared with irradiated cells. (**C**) Effects of knockdown of TRAF4, NOX2, or NOX4 on radiation-induced mRNA expression of ICAM1 were assessed by qRT-PCR. Error bars, ± SEM (n = 3); ^*^ *p* < 0.05 compared with non-irradiated cells, ^**^ *p* < 0.05 compared with irradiated cells. (**D**) Effects of knockdown of TRAF4, NOX2, or NOX4 on secretion of sICAM1 were measured by ELISA in CM of normal lung fibroblasts. Error bars, ± SEM (n = 3); ^*^ *p* < 0.05 compared with non-irradiated cells, ^**^ *p* < 0.05 compared with irradiated cells.

NOX complexes including NOX2 and NOX4 produce superoxide (O_2_^−^), which is rapidly changed to form hydrogen peroxide (H_2_O_2_) by superoxide dismutase and can subsequently freely diffuse through lipid bilayers^23^. It has been reported that endosomal ROS produced by NOXs is important in the redox-dependent activation of NF-κB^19^. Based on these studies, we determined whether TRAF4-NOX-dependent endosomal ROS affected activation of NF-κB. We showed that radiation induced phosphorylation of IκB in cytosol and nuclear localization of NF-κB, which were negatively regulated by treatment with TRAF4, NOX2, or NOX4 siRNA in normal lung fibroblasts (Fig. 4A). In addition, radiation-increased transcriptional activity of NF-κB was significantly reduced by knockdown of TRAF4, NOX2, or NOX4 (Fig. 4B). In a previous microarray study, ICAM1 was identified as a target gene of NF-κB and reported to be overexpressed in CAFs^17^. In addition, secreted soluble ICAM1 (sICAM1) was shown to play a role in cancer invasion and metastasis^24^. In this context, we assessed whether the expression and secretion of ICAM1 were associated with the TRAF4-NOX complex in response to radiation. We found that radiation-induced ICAM1 mRNA expression and sICAM1 secretion were decreased by down-regulation of TRAF4, NOX2, or NOX4 (Fig. 4C,D). These data suggest that endosomal ROS induced by TRAF4-NOX2 or -NOX4 could activate NF-κB signaling and subsequently increase the expression and secretion of ICAM1.

### Secreted sICAM1 in lung fibroblasts enhances aggressiveness of NSCLC cells

**Figure 5:**
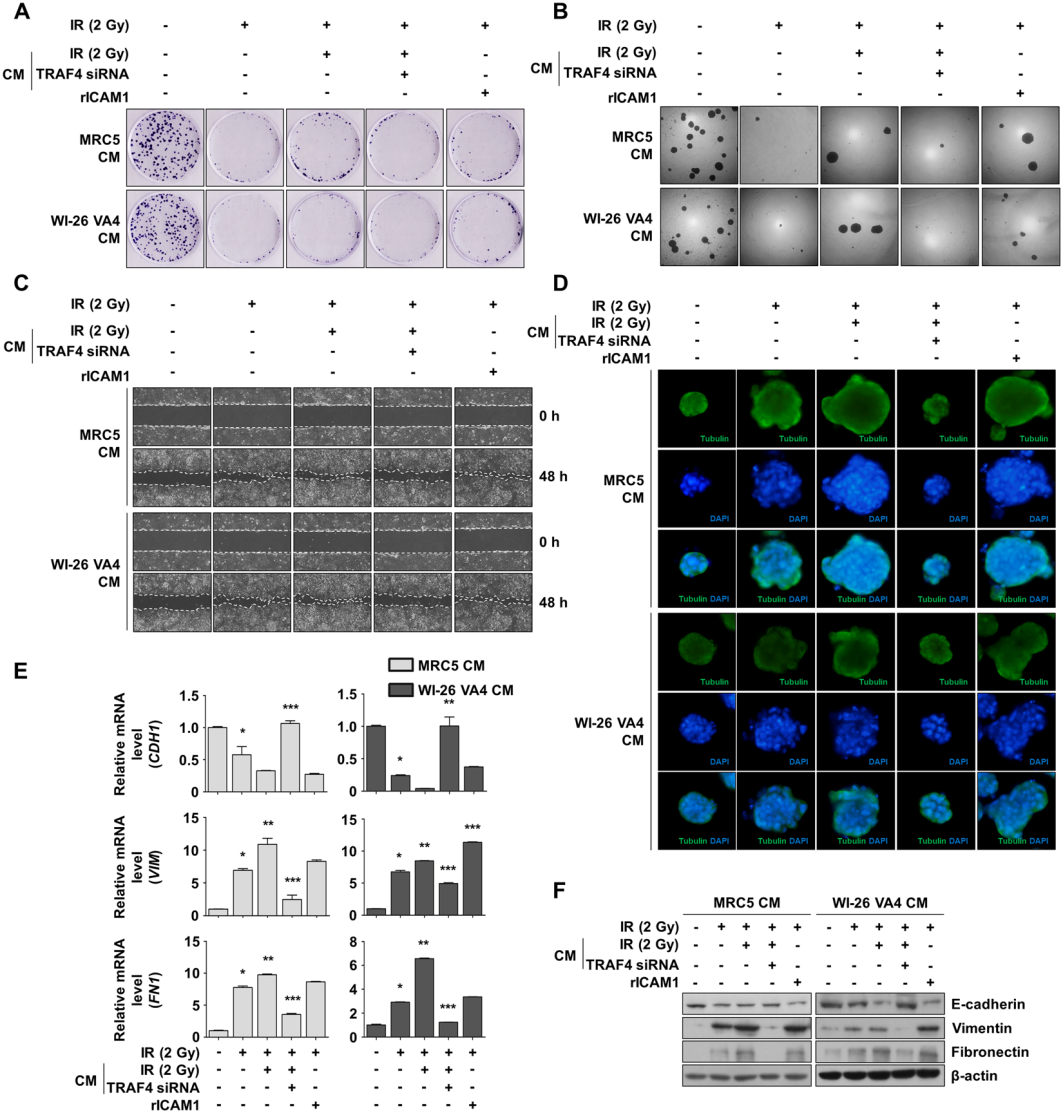
CM collected from irradiated fibroblasts enhanced progression of NSCLC cells. (**A**) The tumor progressive effects of CM on survival of NCI-H460 cells were assessed by colony forming assay. CM was harvested from MRC5 or WI-26 VA4 with designated treatments and NCI-H460 cells were incubated on CM for 7 d. (**B**) Effects of CM on anchorage-independent survival and proliferation of NCI-H460 cells were investigated by soft agar assay. NCI-H460 cells were incubated on CM mixed with agarose for 14 d. (**C**) Effects of CM on invasiveness of NCI-H460 cells were assessed by wound healing assay. Wound closeness was visualized at 48 h after scratching. (**D**) Effects of CM on morphological changes of NCI-H460 cells were visualized in a 3D culture assay. NCI-H460 cells incubated in 3D culture model were stained with IF dyes for tubulin (green) and with DAPI for nucleus (blue). (**E**) The effects of CM on EMT marker of NCI-H460 cells was measured by qRT-PCR. *CDH1* was used for epithelial marker and *VIM* and *FN1* were used for mesenchymal markers. Error bars, ± SEM (n = 3); ^*^ *p* < 0.05 compared with non-irradiated cells, ^**^ *p* < 0.05 compared with irradiated cells, ^***^ *p* < 0.05 compared with cells treated with irradiation and CM collected from irradiated normal fibroblasts. (**F**) Effects of CM on EMT marker of NCI-H460 cells were measured by Western blotting.

Secreted sICAM1 was reported to function as a driving force of tumor progression including increased survival, proliferation, invasiveness, and epithelial-mesenchymal transition (EMT)^25–27^. To determine whether secreted factors of normal lung cells alter the phenotype of NSCLC cells *in vitro*, we first isolated conditioned media (CM) from normal lung fibroblasts and TRAF4-knockdown fibroblasts treated with irradiation. To investigate the effects of sICAM1 secreted from irradiated MRC5 and WI-26 VA4 cells on stimulation of NSCLC cell survival and proliferation, we conducted a colony forming assay and an anchorage-independent soft agar assay. We observed that treatment of CM from irradiated fibroblasts showed increased survival and proliferation of NCI-H460 cells following irradiation. Similar effects were observed following treatment of recombinant ICAM1 (rICAM1), and these effects were diminished by treatment with CM from irradiated TRAF4-knockdown fibroblasts (Fig. 5A,B). To examine the effects of CM and rICAM1 on alteration of invasiveness and EMT in response to radiation, we conducted a wound healing assay and a three-dimensional (3D) culture assay to assess the migration capacity and morphological changes in NCI-H460 cells, respectively. Irradiated NCI-H460 cells treated with CM from irradiated fibroblasts or treated with rICAM1 showed higher motility than those treated with CM from non-irradiated fibroblasts; however, the motility was suppressed by treatment with CM from irradiated TRAF4-knockdown fibroblasts (Fig. 5C). In a 3D culture assay, irradiated NCI-H460 cells treated with CM from irradiated fibroblasts or treated with rICAM1 showed marked morphological changes, which were only reversed by treatment with CM from irradiated TRAF4-knockdown fibroblasts (Fig. 5D). These phenomena were confirmed by analysis of molecular markers involved in EMT. Irradiated NCI-H460 cells showed low expression of an epithelial marker (E-cadherin) and high expression of mesenchymal markers (vimentin and fibronectin) in both mRNA and protein. This expression was intensified by treatment with CM from irradiated fibroblasts or rICAM1 and suppressed by treatment with CM from irradiated TRAF4-knockdown fibroblasts (Fig. 5E,F). These results suggest that radiation-activated TRAF4 in normal lung fibroblasts can modulate the tumor microenvironment and that ICAM1 secreted from irradiated fibroblasts could allow nearby tumor cells to develop aggressively.

### TRAF4-NOX-sICAM1 signaling in normal fibroblasts promotes tumor development of in vivo xenograft mice models

**Figure 6:**
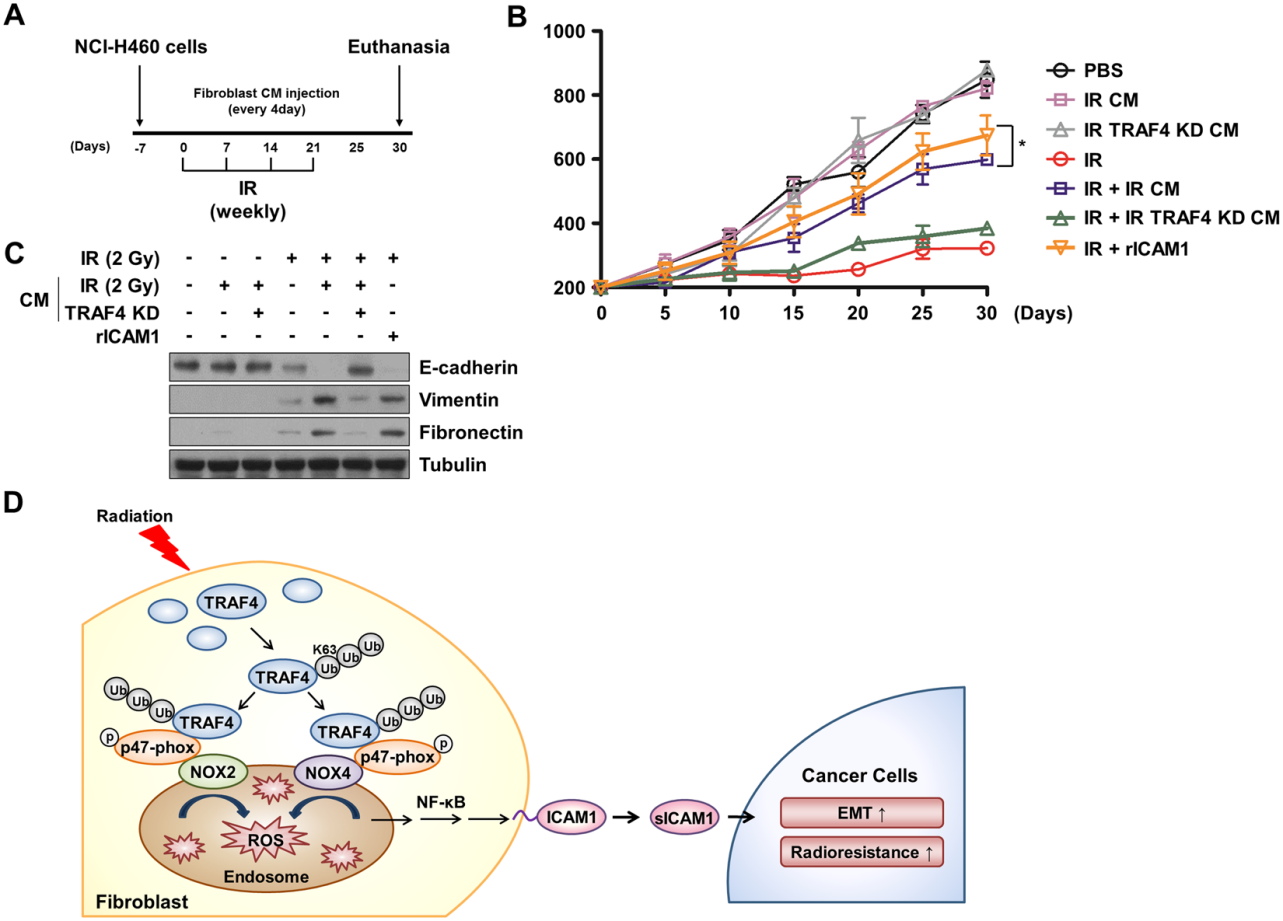
CM from TRAF4-knockdown fibroblasts suppressed *in vivo* tumor progression of NSCLC in a mouse xenograft model. (**A**) Schematic description for generation of mouse xenograft model and treatment of CM. Radiation exposure was locally treated to xenografted tumor sites and each CM was treated for every 4 d, or rICAM1 three times weekly. (**B**) Effects of CM on tumor growth were measured in a mouse xenograft model. Error bars, ± SEM (n = 3); ^*^ *p* < 0.05 compared with irradiated tumor volume. (**C**) *In vivo* effects of CM on EMT marker of tumor were assessed by Western blotting. (**D**) A schematic diagram illustrates how radiation-induced TRAF4 in fibroblasts promotes lung cancer progression. TRAF4 stabilizes NOX complex and increases secretion of sICAM1 which induces EMT and radioresistance of lung cancer cells.

To confirm the involvement of TRAF4 in modulation of the tumor microenvironment, we established an *in vivo* xenograft mouse model. After transplantation of NCI-H460 cells into the flank, mice were subjected to weekly irradiation and treated with CM from irradiated MRC5 cells, CM from irradiated TRAF4-knockdown MRC5 cells every 4 days, or rICAM1 three times weekly (Fig. 6A). As depicted in Fig. 6B, we found that non-irradiated tumors showed no significant changes in tumor growth, regardless of CM treatment. In contrast, irradiated tumors showed increased growth in response to treatment with CM from irradiated MRC5 cells or treatment with rICAM1, while these effects were diminished by treatment with CM from irradiated TRAF4-knockdown MRC5 cells. We also assessed tumor tissue lysates to evaluate protein levels of EMT markers *in vivo*. We found that irradiated tumors showed down-regulated E-cadherin and up-regulated vimentin and fibronectin (Fig. 6C). These changes were enhanced further by treatment with CM from irradiated MRC5 cells or treatment with rICAM1, but were reversed by treatment with CM from irradiated TRAF4-knockdown MRC5 cells. Taken together, these findings indicate that TRAF4 in fibroblasts plays a crucial role in modulation of the tumor microenvironment through up-regulation of sICAM1, leading to stimulation of nearby tumor cell proliferation and EMT.

## Discussion

The tumor microenvironment has been reported to be highly responsible for promotion of invasion, metastasis and therapy-resistance in various cancer types including NSCLC^28, 29^. CAFs are stromal cells that secrete various factors, some of which play a role in ECM remodeling and paracrine signaling associated with formation of the tumor microenvironment^3^. Several studies have indicated that secreted molecules from cancer cells could stimulate normal fibroblasts to be reprogrammed to cancer-supporting cells and CAFs^2, 3^. In this context, we suggest that TRAF4 is a key factor in the transition of normal lung fibroblasts to CAFs by radiation (Fig. 6D). In this study, we showed that TRAF4 interacted with NOX2, NOX4, and phosphorylated p47-phox in normal lung fibroblasts in response to radiation. NOX complexes were localized to endosomes and participated in production of endosomal ROS. Increased endosomal ROS resulted in activation of NF-κB and a subsequent increase in ICAM1 expression. In addition, we found that sICAM1 secreted from normal fibroblasts positively regulated proliferation and EMT of NSCLC cells, both *in vitro* and *in vivo*.

The TRAF family consists of an N-terminal RING domain and a C-terminal TRAF domain with the exceptions of TRAF1 and TRAF7, respectively^30^. The TRAF domain involves TRAF homotrimerization and interacts with upstream regulators and downstream effectors. In this study, we showed that endogenous TRAF4 was detected after IP assay with anti-Flag antibody (Fig. 1). These data indicate that TRAF4 could form a trimer through its TRAF domain, as previously suggested^31^. The RING domain of the TRAF family, which is also found in many E3 ubiquitin ligases, mediates ubiquitination and is associated with a key mechanism in TRAF-dependent signal transduction. A previous study showed that TGF-β did not enhance the poly-ubiquitination of TRAF4 dR mutant, indicating that the RING domain is required for ubiquitination of TRAF4^9^. We also found that a TRAF4 dR mutant did not interact with TβRI/SMURF2 or NOX/p47-phox. In addition, RING domain-containing proteins such as TRAF proteins have shown a high tendency to be ubiquitinated and degraded in a RING domain-dependent manner^32^. CD40-mediated TRAF2 degradation is highly associated with a RING domain of TRAF2, although the RING domain of TRAF2 is not a site for ubiquitination by an E3 ligase^33^. As shown in Fig. 1, expression levels of TRAF4 WT were lower than those of TRAF4 dR, suggesting that a RING domain of TRAF4 is negatively correlated with maintenance of TRAF4 stability. Although further study is required, the results presented herein suggest that the characteristics of TRAF4 are highly associated with the TRAF and RING domains.

NOX4 has an approximate 39% homology with prototypic NOX2 and is a constitutively active form unlike other NOX family members^34, 35^. Although the B-loop between the second and third transmembrane domains of NOX1-NOX4 interacts with a proline-rich region of the C-terminus of p22-phox, each NOX interacts with cytosolic proteins in a different manner^34, 36^. NOX2/p22-phox is activated by binding to phosphorylated p47-phox, p40-phox, p67-phox, and Rac1, while NOX4/p22-phox interacts with DNA polymerase-δ-interacting protein 2 (POLDIP2) to enhance its activity^37^. Structural analysis of NOX4 demonstrated that the NOX4 B-loop has the potential to bind to p47-phox by mimicking the NOX2 B-loop^36^. Our results indicate that NOX4 and NOX2 could form a complex with TRAF4 and p47-phox by radiation (Fig. 1). It was previously suggested that TRAF4 could bind directly to NOX adaptor p47-phox and focal contact scaffold Hic-5, leading to induction of membrane ruffling within the focal complex through Rac1/PAK1 signaling in endothelial cells^18, 38, 39^. In the present study, the interaction of NOX2 and NOX4 with TRAF4/p47-phox was important to the increase of protein stability of NOXs, even though the interaction might not directly affect the activity of NOXs. Consequently, NOX2 and NOX4 could take advantage of protein accumulation in endosomes by avoiding lysosomal degradation associated with autophagy, which is consequently responsible for endosomal ROS escalation in response to radiation.

The location of ROS production is associated with localization of the NOX complexes such as in lipid rafts, endosomes, and the nucleus^39^. We focused on endosomal ROS according to the localization of NOX2 and NOX4 to endosomes in response to radiation (Fig. 2). In a prior investigation, dissociation of NOX2 and p22-phox in the ER resulted in their rapid degradation in a proteasome-dependent manner, which might be regulated by negative regulator of reactive oxygen species (NRROS)^22^. Degradation of NOX4 is also mediated by ubiquitination-dependent proteasomal degradation through Cbl-c and HSP27 signaling^40^. Moreover, it was suggested that a NOX2/p22-phox complex in phagosomal and plasma membranes might undergo lysosomal degradation associated with autophagy^22, 41^. In this study, we found that radiation-activated TRAF4 increased the protein stability of NOX2 and NOX4 by holding in endosomes (Fig. 3). We assumed that sequestered NOX2 and NOX4 in endosomes might have an opportunity to exert their functions such as ROS generation. We showed induction of endosomal ROS for up to 6 h after irradiation (Fig. 2), although ROS are generally produced in the first 30 min after irradiation^42^. We propose that the endosomal ROS might be different from early production of ROS in response to radiation and arise from the functions of TRAF4-NOX complexes in endosomes. These findings indicate that TRAF4 might maintain ROS production and subsequent NF-κB activation by stabilizing NOX complexes. Several studies have reported that NF-κB might be activated in response to irradiation and play a crucial role in survival and EMT induction associated with resistance to radiation in both normal cells and cancer cells^43–45^. Among target genes transcriptionally activated by NF-κB, secretory proteins such as ICAM1 from normal fibroblasts could be highly responsible for constitution of tumor microenvironment. Thus, NF-κB activated by NOX complexes might consequently give an opportunity for normal fibroblasts to survive in response to irradiation and to contribute to promotion of nearby tumor malignancy.

It has been reported that expression of ICAM1 is increased in benign and malignant tumors, and that patients with lung and colorectal cancer show a high level of sICAM1 in blood that is correlated with a poor outcome in disease-free survival^46, 47^. Because sICAM1 retains the extracellular domain, including leukocyte-function associated antigen-1 (LFA-1) binding sites, circulating sICAM-1 could bind to LFA-1 of cytotoxic lymphocytes in blood, consequently allowing cancer cells to escape immune recognition^48^. Although ICAM1 is involved in angiogenesis through regulation of endothelial cell migration^49^, it is unclear whether sICAM directly affects cancer aggressiveness. As shown in Fig. 5, sICAM1 might play a role in the induction of proliferation and EMT in NSCLC cells. In particular, we found intensive proliferation of NSCLC cells treated with irradiation and rICAM1, suggesting that sICAM1 might promote proliferation in surviving cancer cells after irradiation. Thus, through secretion of sICAM1 to the tumor microenvironment, fibroblasts near tumor cells could support advantageous conditions for tumor growth and malignancy with reduced tumor immunity.

The involvement of the tumor microenvironment in tumor progression is widely accepted, and many studies have been conducted reveal the components affecting the tumor microenvironment to improve cancer therapy. In this study, we suggest that post-translational modification of TRAF4 plays a role in allowing fibroblasts to establish a tumor progressive microenvironment. We provide evidence that TRAF4 is an upstream regulator of radiation-induced ROS generation and secretion of sICAM1. Moreover, we showed that TRAF4 knockdown successfully delayed formation of tumor microenvironment. We propose that TRAF4 in fibroblasts could be a potential target to sensitize tumor cells, and that radiotherapy combined with TRAF4 knockdown could be a promising strategy against NSCLC.

## Materials and Methods

### Chemicals, antibodies and reagents

Antibodies specific for TRAF4 (sc-10776), SMURF2 (sc-25511), TβRI (sc-398), SMAD3 (sc-101154), Flag (sc-166355), β-actin (sc-47778), NOX2 (sc-130543), NOX4 (sc-30141), p47-phox (sc-17845), EEA1 (sc-137130), and LAMP1 (sc-5570 and sc-20011) were purchased from Santa Cruz Biotechnology (Santa Cruz, CA, USA). Antibodies specific for GAPDH (MCA4739) and EEA1 (ab2900) were purchased from Bio-Rad (Hercules, CA, USA) and Abcam (Cambridge, MA, USA), respectively. Antibodies specific for p-SMAD3 (9520), p-p38 (9211), p38 (9212), K63-linkage poly ubiquitin (5621) were purchased from Cell signaling Technology (Beverly, MA, USA). Antibody specific for p-p47-phox (orb256707) was purchased from Biorbyt (Riverside, UK). SiRNAs specific for TRAF4 (100153), NOX2 (1038269), and NOX4 (1104575) were purchased from Bioneer (Seoul, Korea). MG132 (C2211) and Chloroquine (C6628) were purchased from Sigma (St. Louis, MO, USA). Recombinant soluble ICAM1 (ADP4-050) was purchased from R&D system (Minneapolis, MN, USA). Dulbecco’s modified eagle medium (DMEM), fetal bovine serum (FBS), penicillin, and streptomycin were acquired from Gibco (Grand Island, NY, USA).

### Cell culture, irradiation, drug treatment, and transient transfection

MRC5 and WI-26 VA4 were purchased from American Type Culture Collection (ATCC, Manassas, VA, USA), authenticated, and maintained in early passages, no more than 6 months after receipt from ATCC. The cells were cultured in DMEM medium containing 10% FBS, 100 U/mL penicillin, and 100 μg/mL streptomycin at 37 °C in 95% air/5% CO_2_. For transient transfection, cells were plated at a density of 6 × 10^5^ cells in 60 mm dishes and incubated for 24 h. The cells were transiently transfected with siRNAs adequate for the experiments. The media was changed with fresh media after 6 h transfection and subsequent treatment or harvested is performed. For treatment, transfected or non-treated cells with 80% confluence were administrated with MG132 (10 μM) dissolved in dimethyl sulfoxide (DMSO) or Chloroquine (100 μM) dissolved in distilled water. After 30 min of incubation, the cells were irradiated with 2 Gy of γ-ray by using a Gamma cell 40 Exactor (Nordion International, Inc. Kanata, Ontario, Canada). The cells were utilized in subsequent experiments after 4 h after irradiation.

### Real-time quantitative RT-PCR (qRT-PCR)

The mRNA levels were determined by qRT-PCR following previous study^50^. Total RNA from cells or from xenografted tumor was isolated by TRIzol® (15596-026, Invitrogen, Carlsbad, CA, USA). The RNA is used to confirm TRAF4, NOX2, NOX4 and ICAM1 mRNA levels after each treatment. To obtain cDNA from isolated mRNA, the isolated mRNA was utilized by reverse transcription (RT) reaction, which was conducted on an ImProm-IITM RT system (A3800, Promega, Madison, WI, USA) following the manufacturer’s protocol. Primers used for mRNA expression are listed in Supplementary Table 1. RT reaction conditions were 25 °C for 5 min, 42 °C for 60 min, 85 °C for 5 min, and 4 °C for overnight. A SYBR Green core reagent kit (4367659, Applied Biosystems, Foster City, CA, USA) and a real-time PCR plate (N8010560, Applied Biosystems) were used for performing qRT-PCR, which was performed by using the Applied Biosystems-7900 HT qRT-PCR instrument (Applied Biosystems). The qRT-PCR conditions were 40 cycles of 15 s at 95 °C and 1 min at 60 °C, which was followed by thermal denaturation. Each mRNA level was measured in triplicate. In addition, each mRNA level was normalized by the GAPDH mRNA level and calculated by using the 2^−ΔΔCT^ method. To simplify data presentation, relative expression values were multiplied by 10^2^.

### Western blotting

After treatments appropriate for each experiment, Western blotting was performed as previously described^51^. Whole cell lysates and tissue lysates were obtained in lysis buffer (20 mM Tris (pH 7.4), 150 mM NaCl, 1% Triton X-100, 0.1% SDS, 0.5% sodium deoxycholate, 10 mM PMSF, 5 μg/mL Leupeptin, 1 μg/mL Aprotinin, and 1 μg/mL Pepstatin A). A BioRad protein assay kit (Bio-Rad) was used to determine protein concentrations in lysates. SDS-PAGE was performed with the protein samples and proteins were transferred to a nitrocellulose membrane. 5% bovine serum albumin (BSA) in TBST (10 mM Tris, 100 mM NaCl, and 0.1% Tween 20) was used for blocking for 1 h at room temperature. Membranes were then incubated with specific primary antibodies at 4 °C overnight and subsequently probed with peroxidase-conjugated secondary antibodies (Enzo Life Sciences, Plymouth Meeting, PA, USA) for 1 h at room temperature. Blots were visualized by using an ECL detection system (Abfrontier, Seoul, Republic of Korea).

### Immunoprecipitation (IP) assay

Interaction between proteins was determined by IP assay following previous study^52^. Cells with treatment adequate for experiments were harvested and whole cell lysates were prepared by using lysis buffer (20 mM Tris (pH 7.4), 2 mM EDTA, 25 mM NaF, 1% Triton X-100, 10 mM PMSF, 5 μg/mL Leupeptin, 1 μg/mL Aprotinin, and 1 μg/mL Pepstatin A). Protein samples were incubated with specific primary antibodies at 4 °C overnight and protein G-agarose beads (Santa Cruz Biotechnology, CA, USA) were applied for IP assay. After washing three times with wash buffer (50 mM Trix (pH 8.0), 150 mM NaCl, 1% NP-40, 0.1% SDS, and 0.5% sodium deoxycholate), immunoprecipitates mixed with SDS sample buffer were boiled and centrifuged. Protein samples were detected by Western blotting.

### Immunocytochemistry

To observe the co-localization of NOX2, NOX4, LAMP1 and EEA1 in MRC5 and WI-26 VA4, immunocytochemistry was performed following a previous study^53^. Cells were cultured on the slide glass (Muto. Pure Chemicals Co. Ltd, Tokyo, Japan) and treated with irradiation, TRAF4 siRNA, or Chloroquine according to the experiment. Cells were fixed in cold acetone for 10 min at −20 °C and washed with cold PBS twice. The cells were blocked with 1% BSA in PBS for 1 h at room temperature and then incubated with primary antibodies at 4 °C overnight. Next, the cells were washed three times and probed with secondary antibodies conjugated with DyLight 488 or DyLight 594 (Thermo Scientific, Hudson, NH, USA), following staining with DAPI. Fluorescence was visualized with an Olympus FV1000IX81 confocal microscope (Olympus Optical, Tokyo, Japan).

### Endosome isolation

Isolation of endosome- and lysosome-enriched fractions was performed by following the flotation-gradient fractionation method described previously^54^. Collected cells were incubated trypsinization and washed twice by centrifugation at 200 × *g* for 5 min with 50 mL of cold homogenization buffer (HB; 250 mM sucrose, 20 mM Hepes and 0.5 mM EGTA, pH 7.0). The obtained cell pellet was resuspended gently in HB and homogenized with a ground glass cell homogenizer. The homogenate was centrifuged at 800 × *g* for 10 min at 4 °C to isolate the cytosolic supernatant. The cytosolic fraction was centrifuged at 50000 × *g* for 5 min at 4 °C to separate mitochondria. The supernatant was subsequently ultra-centrifuged at 34000 rpm for 15 min at 4 °C (SW 40Ti rotor, Beckman Coulter, Brea, CA, USA) to separate the microsomal fraction. The pellet containing lysosomes/endosomes was then resuspended with the same volume of 62% sucrose solution to make a 40.6% sucrose solution. The diluent was transferred to the bottom of a transparent SW40-Ti centrifuge tube (Beckman Coulter) and was overlaid with subsequently 1.5 mL of a 35% sucrose solution, 30% sucrose solution and then 2 mL of a 25% sucrose solution. The tube was then filled with HB and centrifuged at 27000 rpm at 4 °C for 2 h in a SW40-Ti rotor to separate in two different layers. Each fraction was collected by a syringe with a 22 G × 4 inch needle, mixed with SDS sample buffer, boiled, and centrifuged for preparation of protein samples.

### Cytosol/nuclear fractionation

To prepare cytosol extract (CE) and nuclear extract (NE), cells were suspended in buffer A (10 mM HEPES (pH 7.9) 50 mM NaCl, 1 mM DTT, 0.1 mM EDTA, and protease inhibitors) and incubated for 20 min on ice. An equal volume of buffer B (0.1% NP-40 in buffer A) was then added and further incubated for 20 min on ice. Soluble CE and NE pellets were separated by centrifugation at 5000 × *g* for 2 min. The CE was transferred to a new tube and cellular debris was removed via centrifugation at 5000 × *g* for 2 min. NE pellet was washed two times with buffer A and resuspended by using buffer C (10 mM HEPES (pH 7.9), 400 mM NaCl, 1 mM DTT, 1 mM EDTA, and 1 mM EGTA). The nuclear debris in the NE was cleared by centrifugation at 13,000 rpm for 15 min at 4 °C. The CE and NE were mixed with SDS sample buffer, heated, and centrifuged for preparation of protein samples.

### Luciferase assay

NF-κB-specific luciferase reporter assays were conducted to measure the transcriptional activity of NF-κB. Cells (6 × 10^5^) were plated in 6-well plates and grown to 80% confluence. To evaluate NF-κB pathway activation, cells were transiently transfected with 3 μg of NF-κB luciferase reporter gene (NF-κB-Luc) plasmid and dominant negative IκBα (ΔIκBα) plasmid by using Lipofectamine 2000 (Invitrogen, Carlsbad, CA, USA). Following overnight transfection, luciferase reporter gene assays were carried out as described previously^55^.

### Measurement of endosomal ROS

The measurement of endosomal ROS was performed following a method described previously^56^. Subcellular localization of ROS within endosomes was assessed by using OxyBURST Green dihydro-2,4,5,6,7,7-hexafluorofluorescein (H_2_HFF)–BSA (Molecular Probes, Eugene, OR, USA). Working solutions were prepared immediately prior to use by dissolving H_2_HFF-BSA in PBS under nitrogen and protected from light. Cells (3 × 10^5^) were seeded on glass slide and treated with properly according to the experiments. Subsequently, cells were administrated with OxyBurst Green H_2_HFF-BSA at 50 μg/mL for 2 min at 37 °C. Cells were then fixed in 4% paraformaldehyde and visualized by an Olympus FV1000IX81 confocal microscope (Olympus Optical, Tokyo, Japan).

### sICAM1 assessment

Secretion of sICAM-1 in media was measured with ELISA method. Briefly, cells (6 × 10^5^) were seeded in 6-well plates and grown to 80% confluence. Following the transfection of specific siRNA and irradiation, the media was obtained and concentrated by 2-fold with Centricon-10 concentrator (Millipore, Billerica, MA, USA). Concentrated media were applied in an enzyme-linked immunoassay kit (Abcam), according to the manufacturer’s instructions.

### Preparation of CM

Collection of CM in MRC5 and WI-26 VA4 was performed following previous study^57^. Cells were plated at a density of 5 × 10^4^ cells/mL in 100-mm culture dishes, incubated for 24 h, and then irradiated to 2 Gy. At 2.5 days after irradiation, cells were washed with PBS three times, then further incubated in serum-free media without antibiotics for 36 h. CM were collected and centrifuged to remove any residual cells, after which they were filtered through a 0.2 μm syringe filter. Filtered CM was concentrated 10-fold by using a Centricon-10 concentrator (Millipore, Billerica, MA, USA) at 4 °C, then stored at −20 °C. Following CM collection, the number of cells on the dish was determined and the volume of CM used in each experiment was normalized by cell number.

### Colony forming assay

To observe the survival of NCI-H460 cells colony forming assay were utilized following previous study^57^. NCI-H460 cells (400) were seeded in 35-mm dishes and 24 h later were treated with irradiation or normal fibroblast CM. Media were changed after 24 h of treatment and incubated at 37 °C, 5% CO_2_ for 7 d. The cells were fixed with 10% methanol/10% acetic acid and then stained with 1% crystal violet.

### Soft agar assay

For investigation of anchorage-independent survival of the NSCLC cells, soft agar assay were used as previously described^58^. In brief, cells were administrated with irradiation, drugs, or siRNAs and harvested through trypsinization. 35-mm plates were underlaid with 0.5% low melting agarose (Sigma, St. Louis, MO, USA) in RPMI medium and then were overlaid with 0.2% low melting agarose containing 1,000 cells of harvested NCI-H460. Plates were then incubated at 37 °C, 5% CO_2_ for 14 d, and photomicrographs of the colonies were taken at × 100 by using an Olympus IX71 fluorescence microscope (Olympus Optical).

### Wound healing assay

To assess the motility of NCI-H460 cells upon treatment, wound healing assay were used following previous study^59^. In 60-mm dishes, cells with 70% confluency were administrated with the appropriate treatment. After 24 h, the media were changed into RPMI-1640 medium supplemented with 1% FBS and monolayers were scratched by using a 200 μL pipette tip. Cells were then further incubated at 37 °C, 5% CO_2_ for 48 h. Photomicrographs of closed wounds were taken at × 100 by using an Olympus IX71 fluorescence microscope (Olympus Optical).

### 3D culture

3D cell culture was utilized to observe morphological changes in NCI-H460 cells upon extracellular environments following previous study^60^. Matrigel (BD Biosciences, Bedford, MA, USA) was thawed at 4 °C overnight. NCI-H460 cells were harvested through trypsinization and suspended in serum-free RPMI medium in density of 25,000 cells/mL. 200 μl of cells were mixed with 200 μl of 4% Matrigel in RPMI medium and the mixture was dispensed into an each well of eight-well chambered glass slide (Nunc, Napervile, IL, USA). After 3 d of attachment of the cells at 37 °C and 5% CO_2_, cells were administrated with CM obtained from MRC5 or WI-26VA4 cells that had been treated with irradiation, rICAM1, or siRNA specific for TRAF4 and incubated for 24 h. Cells were fixed in 2% paraformaldehyde for 20 min, permeabilized in 0.5% Triton X-100 for 10 min, then washed with cold PBS (NaCl 137 mM, KCl 2.7 mM, Na_2_HPO_4_ 10 mM and KH_2_PO_4_ 1.8 mM). After blocking with 1% BSA in PBS for 1 h at room temperature, the cells were incubated overnight with anti-Tubulin antibodies at 4 °C. Next, cells were incubated with secondary antibodies conjugated with DyLight 488 (Thermo Scientific, Hudson, NH, USA) and counterstained with 4′,6-diamidino-2-phenylindole (Sigma). Fluoresence was visualized with an Olympus FV1000IX81 confocal microscope (Olympus Optical).

### Animal care protocol

The animal protocols were approved by Institutional Animal Care and Use Committee of Pusan National University (Busan, South Korea), and performed following the NIH Guide for the Care and Use of Laboratory Animals. Mice were randomly allocated and housed individually or in groups of up to five in sterile cages. Animals were maintained in animal care facilities at 23 ± 1 °C and under a 12 h light/dark cycle, and were quarantined for 1 week prior to study. The animals were fed water and a standard mouse chow diet *ad libitum*.

### Tumor xenografts in nude mice

Tumor xenografts in nude mice were performed following the previous study^61^. Six-week-old male BALB/c athymic nude mice (Central Lab Animals, Seoul, South Korea) were randomly separated into three per groups for the experiments. The animals were injected in the flank with 2 × 10^6^ NCI-H460 cells adapted to normal media, MRC5 CM or TRAF4 knockdown MRC5 CM in the flank. Tumors were allowed to develop and CM or recombinant soluble ICAM1 (40 μg/kg body weight) adequate for each group was injected into the tumor every four days during 30 d of incubation by using an insulin syringe. Irradiation was administrated to the animals at 10 Gy once a week for 30 d. To calculate tumor volumes, tumor length (L) and width (l) were measured with a caliper and the formula (L × l^2^)/2 was used. At the end of the treatment period, animals were euthanized and the tumors were used for subsequent experiments.

### Statistical analysis

All numeric data are presented as the mean and standard deviation (SD) or standard error of mean (SEM) obtained from at least three independent experiments. A one-way ANOVA was used for ranked data followed by Tukey’s honestly significant difference test, and a two-way ANOVA was used for ranked data followed by a Bonferroni post-test. All statistical analyses were performed by using Prism 4 software (GraphPad Software, San Diego, CA, USA). A *p*-value < 0.05 was considered to indicate statistical significance.

## Electronic supplementary material

Supplementary Information
