# Supplementary material for: TRAF4 promotes lung cancer aggressiveness by modulating tumor microenvironment in normal fibroblasts
Source: Sci Rep. 2017 Aug 21;7:8923. doi: 10.1038/s41598-017-09447-z (PMC5566719; doi:10.1038/s41598-017-09447-z)
Supplement: Supplementary file 1 — Supplementary Information [file 41598_2017_9447_MOESM1_ESM.pdf]

TRAF4 promotes lung cancer aggressiveness by modulating tumor microenvironment in normal fibroblasts

EunGi Kim[1,*], Wanyeon Kim[2,a,*], Sungmin Lee[1], Jahyun Chun[1], JiHoon Kang[1], Gaeul Park[1], IkJoon Han[2], Hee Jung Yang[1], HyeSook Youn[3], and BuHyun Youn[1,2]

[1]Department of Integrated Biological Science, Pusan National University, Busan 46241, Republic of Korea; [2]Department of Biological Sciences, Pusan National University, Busan 46241, Republic of Korea; [3]Department of Integrative Bioscience and Biotechnology, Sejong University, Seoul 05006, Republic of Korea

[a]Current address: Department of Biology Education, Korea National University of Education, Cheongju 28173, Republic of Korea

[*]E. Kim and W. Kim contributed equally to this work

Corresponding author: Prof. BuHyun Youn, Department of Biological Sciences, Pusan National University, Busandaehak-ro 63beon-gil, Geumjeong-gu, Busan 46241, Republic of Korea

E-mail: bhyoun72@pusan.ac.kr; Tel: 82-51-510-2264; Fax: 82-51-581-2962

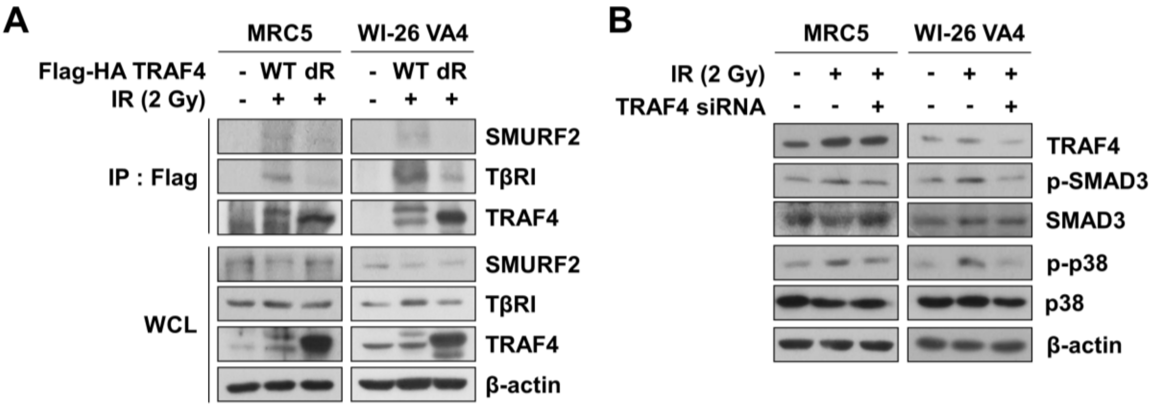

Supplementary Figure 1. K63-linkage poly-ubiquitinated TRAF4 promotes TβRI signaling in response to radiation. (**A**) Binding of radiation-induced TRAF4 to TβRI and SMURF2 was assessed by IP assay. After transfection with Flag-HA-TRAF4 WT or Flag-HA-TRAF4 dR mutant, cell lysates were immunoprecipitated with anti-Flag antibody followed by Western blotting in response to radiation. (**B**) Effects of TRAF4 knockdown on TβRI signaling pathway were investigated by Western blotting.

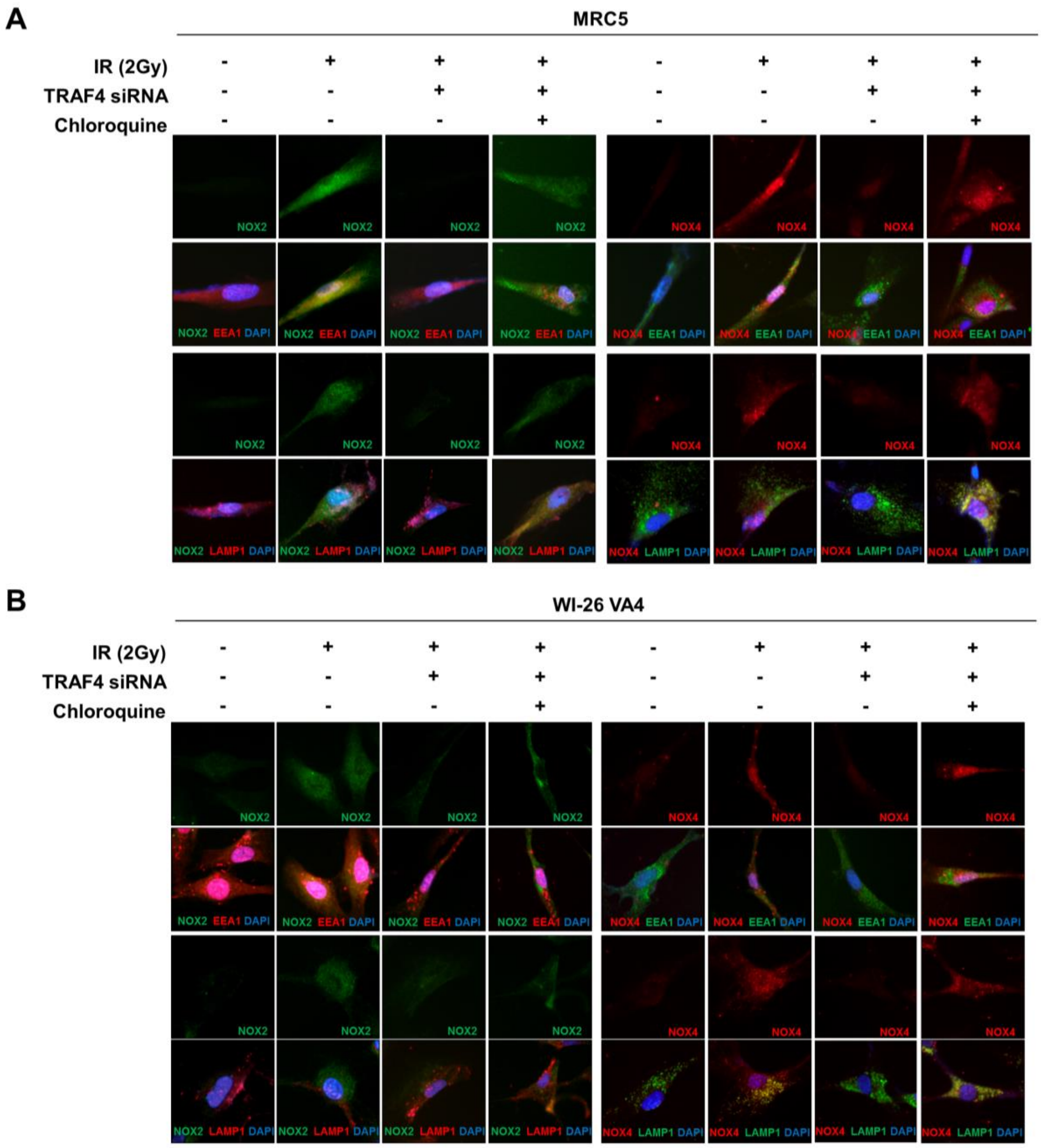

Supplementary Figure 2. TRAF4 stabilized NOX2 and NOX4 through regulation of lysosomal degradation in response to radiation. (**A**) Chloroquine-induced stabilization of NOX2 and NOX4 in endosomes or lysosomes was visualized by immunocytochemistry in MRC5 cells. Cells were stained with specific antibodies after treatment of Chloroquine and irradiation in TRAF4-knockdown cells. EEA1 and LAMP1 were used as biomarkers for endosomes and lysosomes, respectively. (**B**) Chloroquine-induced stabilization of NOX2 and NOX4 in endosomes or lysosomes was visualized by IF analysis in WI-26 VA4 cells. Cells were stained with specific antibodies after Chloroquine treatment and irradiation in TRAF4-knockdown cells.

Supplementary Table 1. Primers for determining expression levels of several genes

Gene name	Forward primer	Reverse primer
TRAF4	5’-GCT GCA TCC ACA GTG AGG AGG G-3’	5’-CTC AGA GGT GGC ATG CTG GGC C-3’
NOX2	5’-TGT TCA GCT ATG AGG TGG TGA-3’	5’-TCA GAT TGG TGG CGT TAT TG-3’
NOX4	5’-CTC AGC GGA ATC AAT CAG CTG TG -3’	5’-AGA GGA ACA CGA CAA TCA GCC TTA G-3’
ICAM1	5’-AAA AGT CAT CCT GCC CCG GG-3’	5’-AGG GCA GTT TGA ATA GCA C-3’
CDH1	5’-GGA TTG CAA ATT CCT GCC ATT C-3’	5’-AAC GTT GTC CCG GGT GTC A-3’
VIM	5’-GAC AAT GCG TCT CTG GCA CGT CTT-3’	5’-TCC TCC GCC TCC TGC AGG TTC TT-3’
FN1	5´-TGA CCT TTT CTG GCT CGT CT-3´	5’-GTT CAG CAC AAA GGG CTC TC -3’
GAPDH	5’-TGT TGC CAT CAA TGA CCC CTT-3’	5’-CTC CAC GAC GTA CTC AGC G-3’
